# Establishment and molecular characterization of HCB-541, a novel and aggressive human cutaneous squamous cell carcinoma cell line

**DOI:** 10.1007/s13577-024-01054-1

**Published:** 2024-04-03

**Authors:** Ana Carolina Laus, Izabela Natalia Faria Gomes, Aline Larissa Virginio da Silva, Luciane Sussuchi da Silva, Mirella Baroni Milan, Silvia AparecidaTeixeira, Ana Carolina Baptista Moreno Martin, Letícia do Nascimento Braga Pereira, Carlos Eduardo Barbosa de Carvalho, Camila Souza Crovador, Flávia Escremin de Paula, Flávia Caroline Nascimento, Helder Teixeira de Freitas, Vinicius de Lima Vazquez, Rui Manuel Reis, Renato José da Silva-Oliveira

**Affiliations:** 1 Molecular Oncology Research Center, Barretos Cancer Hospital, Antenor Duarte Villela, 1331, Barretos, São Paulo, Zip Code: 14784 400 Brazil; 2 Department of Surgery of Melanoma and Sarcoma, Barretos Cancer Hospital, São Paulo, Brazil; 3 Life and Health Sciences Research Institute (ICVS) Medical School, University of Minho, Braga, Portugal; 4 ICVS/3B’s-PT Government Associate Laboratory, Braga/Guimarães, Portugal; 5 Barretos School of Health Sciences, Dr. Paulo Prata-FACISB, Barretos, São Paulo, Brazil

**Keywords:** Cutaneous squamous cell carcinoma, Molecular profile, Cell line establishment, In vivo model

## Abstract

Cutaneous squamous cell carcinoma (cSCC) is a common type of skin cancer that can result in significant morbidity, although it is usually well-managed and rarely metastasizes. However, the lack of commercially available cSCC cell lines hinders our understanding of this disease. This study aims to establish and characterize a new metastatic cSCC cell line derived from a Brazilian patient. A tumor biopsy was taken from a metastatic cSCC patient, immortalized, and named HCB-541 after several passages. The cytokeratin expression profile, karyotypic alterations, mutational analysis, mRNA and protein differential expression, tumorigenic capacity in xenograft models, and drug sensitivity were analyzed. The HCB-541 cell line showed a doubling time between 20 and 30 h and high tumorigenic capacity in the xenograft mouse model. The HCB-541 cell line showed hypodiploid and hypotetraploidy populations. We found pathogenic mutations in *TP53* p.(Arg248Leu), *HRAS* (Gln61His) and *TERT* promoter (C228T) and high-level microsatellite instability (MSI-H) in both tumor and cell line. We observed 37 cancer-related genes differentially expressed when compared with HACAT control cells. The HCB-541 cells exhibited high phosphorylated levels of EGFR, AXL, Tie, FGFR, and ROR2, and high sensitivity to cisplatin, carboplatin, and EGFR inhibitors. Our study successfully established HCB-541, a new cSCC cell line that could be useful as a valuable biological model for understanding the biology and therapy of metastatic skin cancer.

**Supplementary Information:**

The online version contains supplementary material available at 10.1007/s13577-024-01054-1.

## Introduction

Cutaneous squamous cell carcinoma (cSCC) is a prevalent type of nonmelanoma skin cancer, accounting for 20% of all skin cancer cases [1]. Its incidence is increasing in most white populations globally [1]. Depending on the origin of the tumor, cSCC can be classified as cutaneous or invasive. Cutaneous cSCCs arise from keratinocyte proliferation, while invasive cSCCs arise from intraepidermal dysplasia [2]. The primary risk factors for cSCC development include exposure to ultraviolet radiation, chemical carcinogens, immunodeficiency, chronic inflammation, and precursor pre-malignant conditions, including Bowen’s disease, actinic keratosis, and keratoacanthomas [3–5].

The European guideline categorizes cSCCs as primary or advanced, depending on the extent of tumor spread, and primary cSCCs are typically non-metastatic and easily treatable, whereas advanced cSCCs are classified as locally advanced or metastatic. In the early stages of development, cSCCs tend to be indolent with low metastasis rates, resulting in a better prognosis with 5-year cure rates greater than 90% [6]. For small tumors (≤ 1 cm in diameter) with defined primary lesions, effective treatments such as electrodesiccation, curettage, excision, or cryosurgery have shown up to 90% success rates [5]. However, tumors with high metastatic risk and potential aggressiveness require prophylactic irradiation of the regional lymph nodes after surgery, as they become highly aggressive when recurring (8). Advanced cSCCs have a worse prognosis, with 10-year survival rates of less than 20% for patients with regional lymph and less than 10% for those with distant metastases, while approximately 90% of patients with distant metastases had died due to the disease within five years [7, 8].

Understanding the complex molecular profile of cSCC is crucial for developing novel and effective therapies, particularly for the rare metastatic cSCC [9]. In vitro and in vivo cellular models are essential tools for discovering better treatments. However, the limited availability of well-characterized cSCC lines for preclinical experimentation and drug screening presents a challenge [11]. Currently, the A431 cell line derived from the vulva [12] is the only commercially available cSCC line in the American Type Culture Collection (ATCC) and European Collection of Authenticated Cell Cultures (ECACC) cell banks. Recently, a patient-derived cutaneous squamous cell carcinoma (cSCC) xenograft (PDX) was generated, replicating both the histological and genetic attributes of the primary tumor, and exhibiting a response profile to eribulin and cisplatin, underscoring the critical utility of establishing primary lineages for delineating novel therapeutic strategies [10].

Therefore, this study aimed to establish and characterize a new advanced cSCC cell line that preserves the biological properties of metastatic cancer cells and can be used for future investigations of their biology and novel effective anti-cancer agents.

## Materials and methods

### Clinical presentation

A 67-year-old male patient had undergone excision of cutaneous epidermoid carcinoma on his anterior chest the year before and sought medical care at the surgical oncology outpatient clinic at Barretos Cancer Hospital, Brazil, with a persistent tumor in the left armpit (Supplementary Fig. 1). The patient had a history of acute myocardial infarction, coronary stent placement, and diabetes. A tumor biopsy at Barretos Cancer Hospital revealed a cutaneous squamous cell carcinoma (cSCC), and the patient underwent surgery and received postoperative carboplatin, taxane, and radiation therapy. Unfortunately, the patient experienced a recurrence shortly after treatment and was deemed no longer treatable, ultimately resulting in his death.

### HCB-541 cell line establishment

Surgical specimens were collected for cell cultivation immediately after surgery and maintained in Dulbecco’s Phosphate Buffered Saline solution until processing begins as previously reported [13]. Biopsy tumor was fragmented into small pieces with a surgical scalpel in a petri dish. Then, it was incubated in 0.5 ml of accutase/trypsin enzymatic solution for total tissue dissociation, at 37 °C for 30 min. After dissociation, the cell solution was incubated in DMEM medium supplemented with 10% Fetal Bovine Serum (SBF) and 1% penicillin and streptomycin to complete enzyme inactivation. Throughout the study, experiments were consistently conducted using aliquots from this identical FBS batch (210625 K) to ensure quality and minimize variations in component composition. Cells were cultivated in DMEM medium supplemented with 1% penicillin/streptomycin, 10% FBS and maintained in culture flask at 37 °C, 5% CO_2_. Primary cell line was named HCB-541, and stock vials were frozen in different passage numbers to report the protein expression change during establishment processes, and for DNA and RNA analysis at the 15th passage.

### Morphological and doubling time characterization

To assess the morphologic profile, after continuous passages (> 30), the HCB-541 cell line was seeded in plates and cultivated in DMEM supplement with 1% P/S and 10% SFB at 37 °C, 5% CO2. Cells were photographed under an optical microscope at × 100 magnification (Olympus XT01), until complete 90% of total confluence. Doubling time values were obtained using xCELLigence real-time cell analysis (Agilent Technologies, Inc), according to the manufacturer’s instructions. Initially, 3.5 × 10^3^ or 7 × 10^3^ cells were plated into E-plate (Agilent Technologies, Inc) and were cultivated in DMEM medium supplemented with 1% P/S and 10% for 72 h. The cell index was calculated using a specific RCCA® software analysis.

### Tumorigenicity of HCB-541 cell line in NOG-SCID mice

To assess the tumorigenicity of HCB-541, subcutaneous injections of either 1 × 10^6^ or 3 × 10^6^ cells mixed with Matrigel® at a 1:1 (v/v) ratio were administered into the right flank of 8-week-old female NSG mice (NOD.Cg-Prkd^cscid^ Il2rg^tm1Wjl^/SzJ, The Jackson Laboratory, USA), as previously described [14]. Tumor size was measured weekly with calypter, and volume was calculated using the formula: volume = length × width2/2. After two weeks of transplantation, tumors were excised surgically, measured, and subjected to morphological evaluation using hematoxylin and eosin staining and immnunohistochemistry (IHC) analysis. Tumors were fixed in a 4% paraformaldehyde, and paraffin-embedded sections were prepared using standard procedures for histological analysis. The mice used in this study were maintained under SPF conditions, on a 12-h light/dark cycle, and received sterile food and water ad libitum.

### Immunophenotypic characterization

We conducted an immunocytochemistry (ICC) analysis of the HCB-541 cells and compared with controls A431 (human vulva epidermoid carcinoma cell line) (RRID:CVCL_0037), from ATCC (ID number: CRL-1555) and HACAT (immortalized normal human keratinocyte cell line)(RRID:CVCL_0038) from BCRJ (ID number: 0341). Cells were seeded in a chamber slide with 8 wells on a glass slide overnight and then fixed with paraformaldehyde in DPBS for 5 min. After the fixation step, cells were permeabilized with Triton-X 100 0.25% in DPBS for 10 min. Protein blocking was performed with Lab Vision™ UltraVision™ (Thermo Scientific), according to the manufacturer's protocol. Primary antibodies ready to use anti-vimentin (RRID:AB_2722716), anti-KI67 (RRID:AB_2250503), anti-cytokeratin 20 (RRID:AB_563800), anti-cytokeratin AE1/AE3 (RRID:AB_1587224)) anti-cytokeratin 8/18 (RRID:AB_563833), anti-cytokeratin 17 (RRID:AB_2133033)) were incubated overnight. After the incubation, the slides were in biotinylated goat polyvalent antibody for 10 min, followed by washing and subsequently incubated with streptavidin peroxidase, stained with DAB chromogen and counterstaining with hematoxylin eosin. Finally, the cells were photographed using an optical microscope Olympus XT01.

Immunohistochemistry expression of CK 5/6 (RRID:AB_563822), p63 (RRID:AB_10582857), p40 (RRID:AB_3073535) and p10 (RRID:AB_2549617) were performed in tumor tissue biopsy, HCB-541 cell line, both in formalin-fixed paraffin-embedded (FFPE) sections using BenchMark ULTRA IHC/ISH System (Roche) automated staining platform.

### Cell line authentication by short tandem repeat (STR) profiling analysis and mycoplasma contamination test

STR analysis was performed using DNA from the cell line and FFPE tumor biopsy as previously described for authenticity confirmation [15]. The mycoplasma contamination test was carried out in DNA from the cell line using MycoAlert Mycoplasma Detection Kit (Lonza, Swiss), according to the manufacturer's protocol, in different moments throughout the time it was in culture.

### DNA and RNA isolation

DNA was isolated from the patient's formalin-fixed and paraffin-embedded (FFPE) tumor biopsy, using QIAamp DNA Micro Kit (Qiagen) as previously reported [16]. DNA from the HCB-541 cell line was isolated using Biopur Mini Spin Plus 250 Extraction kit (Biopur), both according to the manufacturer's recommendations. All samples were quantified by NanoDrop 2000 System (Thermo Scientific) and stored at −20 °C. RNA was isolated from HCB-541 and HACAT (immortalized normal human keratinocyte) cells using RecoverAll Total Nucleic Acid Isolation kit (Invitrogen), according to the manufacturer's recommendations. Samples were quantified by NanoDrop 2000 System (Thermo Scientific) and Qubit 2.0 Fluorometer (Life Technologies), and stored at −80 °C.

### Cellular viability assay (MTS)

Cell viability was determined 72 h after drug exposure, using the colorimetric CellTiter 96® AQueous One Solution Cell Proliferation Assay (Promega, Madison, WI), according to the manufacturer’s instructions and as previously reported [15]. To assess cytotoxicity, a total of 5 × 10^3^ were plated in 96 well plates in DMEM with 10% FBS and 1% allowed adherence overnight. Then, cells were treated with increased concentrations of pharmaceutical agents and reader (Thermo Scientific, Finland), at 490 nm. Results were normalized with DMSO control values. To calculate IC_50_ values the GraphPad Prism software (Version 9.0) was used to evaluate a nonlinear regression curve. All experiments were performed in triplicates at least three times.

### Western blot and reverse phase protein arrays (RPPA)

Protein lysate of HCB-541 and A431 (epidermoid cancer cell line) cells were used to perform western blot analysis. Both cells were rinsed in DPBS and after lysed in lysis buffer following our group protocol [17] and were incubated overnight with primary antibody: pEGFR-Tyr1068 (D7A5), t44/42 MAPK (137F5); p.p44/42 MAPK-Thr202/Tyr204 (D13.14.4E); AKT(pan) (C67E7); pAKT-Ser473 (D9E); phosphoP53-Ser15 (82530), phosphor-P53-Ser46 (2521), ROR-2 (88639) FGFR1 (ab76464) and β- actin (3700) were used. Both primary antibodies from Cell signaling were diluted in TBS-T at 1:1000. Membranes were incubated with anti-rabbit secondary antibody Anti-rabbit (7074) at dilution 1:5000. Chemiluminescent signals were detected by ECL in automatic ImageQuant mini LAS4000 (GE Healthcare Protein array was Human Phospho-Mitogen-activated (ARY002B; R&D Systems, MN) and Human Phospho-RTK Array (ARY001B; R&D Systems, MN) were conducted according to the manufacturers' instructions). All the experiments were performed three times.

### Pharmacological agents

Small-molecules inhibitors Afatinib (Cat.N° S1011); Allitinib (Cat.N° S2185); Lapatinib (Cat.N° S2111); Erlotinib (Cat.N° S1023) were purchased from Selleck Chemicals (Houston, TX). Carboplatin (Cat.N° C2538), Cisplatin (Cat.N° 15,663-27-1) and 5-Fluoracil (Cat.N° 51-21-8) were purchased from Sigma Aldrich (Sigma-Aldrich, USA) and Cetuximab was purchased from Merck and were diluted in DMSO at 10 mM and stored at 20 °C for posterior use. Carboplatin was dissolved in distilled water and Cetuximab is ready to use. DMSO or water were used as a negative control in all cytotoxicity experiments. Details of concentrations, doses and exposition time are explained in supplementary Table 1.

**Table 1:** Mutation profile of both tumor biopsy and HCB-541 cell line

Sample	Gene	Mutation type	VAF % (cell line/tumor)	Exon	Protein	Franklin AMP classification	Varsome AMP classification	ClinVar classification
Cell line and tumor	FGFR3	SNV	50.60/41.00	9	p.(Phe384Leu)	Tier IV	Tier IV	Benign/likely benign
Cell line and tumor	HRAS	INDEL	69.80/53.00	5	p.(Arg61Glyfs*32)	Not found	Tier III	Not found
Cell line and tumor	HRAS	SNV	32.30/25.70	3	p.(Arg97Met)	Tier III	Tier III	Not reported
Cell line and tumor	HRAS	SNV	66.20/58.50	3	p.(Gln61His)	Tier III	Tier III	Likely pathogenic
Cell line and tumor	KIT	SNV	41.40/48.30	10	p.(Met541Leu)	Tier III	Tier III	Benign/likely benign
Cell line and tumor	MYC	SNV	63.20/63.50	2	p.(Asn26Ser)	Tier IV	Tier IV	Uncertain significance
Cell line and tumor	NOTCH1	SNV	48.80/33.60	34	p.(Glu2071Lys)	Tier III	Tier III	Not found
Cell line and tumor	PTEN	SNV	97.30/100.00	2	p.(Cys65Ser)	Tier IV	Tier IV	Not found
Cell line and tumor	RAF1	SNV	99.70/76.50	15	p.(Ser549Phe)	Tier III	Tier III	Not found
Cell line and tumor	TERT	SNV	47.90/39.20	UTR (1,295,228)	Non coding	Tier III	Not reported	Pathogenic
Cell line and tumor	TP53	SNV	99.70/65.80	7	p.(Arg248Leu)	Tier III	Tier II	Pathogenic
Only tumor	DICER1	SNV	---/28.60	26	p.(Lys1844Asn)	Tier III	Tier IV	Not found
Only tumor	ESR1	SNV	---/12.50	5	p.(His398Arg)	Tier II	Tier III	Not reported
Only tumor	KIT	INDEL	---/14.30	17	p.(Lys807_Ile808delinsAsnLeu)	Not found	Tier II	Not reported
Only tumor	KIT	INDEL	---/21.80	18	p.(Asn843_Cys844delinsLysArg)	Tier III	Tier III	Not reported
Only tumor	KIT	INDEL	---/20.80	18	p.(Phe848Val)	Tier III	Tier III	Not reported
Only tumor	KIT	SNV	---/12.60	18	p.(Ser850Asn)	Tier III	Tier II	Not reported
Only tumor	PDGFRA	SNV	---/15.50	12	p.(Tyr574His)	Tier III	Tier III	Not found
Only tumor	RAF1	SNV	---/10.80	14	p.(Thr506Ala)	Tier III	Tier III	Not found
Only cell line	NOTCH1	INDEL	50.10/---	34	p.(?)	Tier III	Tier III	Not found

*Tier II* Potential Clinical Significance, *Tier III* Unknown Clinical Significance, *Tier IV* Benign or Likely Benign; *SNV* Single Nucleotide Variant, *INDEL* Insertion/Deletion

### Chromosome preparation and GTG-banding

The karyotype analysis was performed as described previously [18]. HCB-541 cell line at the fifteenth passage was incubated with 120 ng/ml colcemid (Life Technologies-Gibco) for 125 min. The cells were harvested by treatment with 0.025% trypsin EDTA, suspended in KCl 0.075 M solution at room temperature for 5 min, and fixed with methanol/acetic acid (3:1) five times. To examine the chromosomal distribution of constitutive heterochromatin, GTG-banding was performed using the standard trypsin/Giemsa method [19]. The karyotype analysis of HCB-541 cells was done on twenty metaphases.

### Mutational and fusion analysis and clinical significance variants classification

Mutations from the patient's FFPE tumor biopsy and HCB-541 cell line were evaluated by Next Generation Sequencing (NGS), using the Custom Cancer Solution cSTS kit (SOPHiA Genetics). This 48-gene panel plus microsatellite instability (MSI), includes all protein-coding exons for all transcripts of 8 genes, as well as a selection of protein-coding exons of 40 genes with ± 10 bp flanking into the intron and, if applicable, including 5’ UTR exons that contain coding sequence (Supplementary Table 2). No normal DNA tissue (blood or adjacent normal skin) was available for germline mutation analysis. Genetic variants were evaluated in both HCB-541 cell line and the primary tumor. Moreover, the presence of gene rearrangements was performed on the HCB-541 cell line, using also the Custom Cancer Solution (cSTS) kit (SOPHiA GENETICS, Switzerland) according to the manufacturer’s protocol. The somatic RNA panel for fusion research includes 16 genes: *ALK, BRAF, EGFR, FGFR1, FGFR2, FGFR3, FGFR4, MET* exon 14 skipping, *NTRK1, NTRK2, NTRK3, RET, ROS, ERG, PPARG* and *NRG1*.

Briefly, from 20 ng of DNA, fragments were generated using an enzymatic fragmentation step. The three subsequent enzymatic steps, end-repair, A-tailing, and ligation to Illumina adapters, were performed to produce NGS libraries. A capture-based target enrichment was carried out on the pooled libraries. The quantitation of the final pool of libraries was performed using Qubit dsDNA HS fluorimetric assays (ThermoFisher). Quality control of fragment size was assessed using DNA ScreenTape analysis 4150 TapeStation system (Agilent). Sequencing was achieved with the final library concentration of 10 pM onto a 600-cycle format V3 flow-cell, via Illumina MiSeq platform Illumina. Data analysis was performed to detect Single Nucleotide Variants (SNVs), insertions/deletions (INDELs), and Copy Number Alterations (CNAs). Sequencing FASTQ data were analyzed by the Sophia DDM® platform (SOPHiA Genetics). For gene fusions, 200 ng of RNA was used to convert it into cDNA, and library construction followed the RNA target Oncology solution (ROS) kit by SOPHiA GENETICS, Switzerland, as per the manufacturer protocol. DNA fragments were generated through enzymatic steps, including end-repair, A-tailing, and ligation to Illumina adapters, to create NGS libraries. A capture-based target enrichment was applied to the pooled libraries. The final library pool was quantified using Qubit dsDNA HS fluorimetric assays from Life Technologies, USA. Quality control for fragment size was assessed using DNA ScreenTape analysis with the 4150 TapeStation. Data analysis was performed to detect fusion genes. Sequencing FASTQ data were analyzed by the Sophia DDM® platform (Sophia Genetics, Switzerland).

Selected variants were evaluated according to clinical significance using Varsome [20], Franklin (http://franklin.genoox.com) and ClinVar databases [21], considering evidence of skin squamous cell carcinoma (Table 1).

*TERT* hotspot promoter mutations were also validated by droplet digital PCR (ddpCR). To detected *TERTp* mutations was used 10 ng of DNA, 2X ddPCR Supermix for Probes (No dUTP) (Bio-Rad, Hercules, CA, USA), 5 M Betaine (Sigma), 80 mM EDTA (Sigma), 10U/ul CviQI enzyme (ThermoFisher) and the commercial assay TERT C228T: ddPCR™ Expert Design Assay: TERT C228T_113, Human, Homo sapiens (Bio Rad), following the manufacturer's protocol. Droplets were generated using a droplet generator (Bio‐Rad). The PCR cycling parameters included 10 min at 95 °C, 50 cycles at 96 °C for 30 s and 61 °C for 30 s, and one cycle at 98 °C for 10 min followed by a 4 °C hold. Droplet fluorescence was assessed using a droplet reader (Bio-Rad). Analysis of the ddPCR data for allele calling and the calculation of absolute copy numbers were performed using QuantaSoft software, version 1.7.4 (Bio‐Rad).

### Molecular microsatellite instability (MSI)

MSI evaluation was also performed by multiplex PCR using 6 quasi-monomorphic mononucleotide repeat markers (BAT25, BAT26, NR21, NR24, NR27, HSP110) as described by Berardinelli et al. [22] PCR was performed using Qiagen Multiplex PCR Kit (Qiagen) with 50 ng of DNA and the following thermocycling conditions: 15 min at 95 °C; 40 cycles of 95 °C for 30 s, 55 °C for 90 s and 72 °C for 30 s; and a final extension at 72 °C for 40 min. PCR products were then submitted to capillary electrophoresis on an ABI 3500XL Genetic Analyzer (ThermoFisher) according to the manufacturer’s instructions. The results were analyzed using GeneMapper v4.1 software (ThermoFisher) to measure the fragment length in base pairs. The classification of instability is determined by the number of markers out of the quasi-monomorphic variation range (QMVR), being 2 or more markers classified as MSI-H (MSI-High), only one marker classified as MSI-L (MSI-Low) and cases without markers out of QMVR classified as MSS (Microsatellite stability), as previously reported [22].

### Droplet digital PCR (ddPCR) detection for human papilloma virus (HPV)

The presence of HPV 16 and 18 was evaluated in the patient's tumor biopsy and HCB-541 cells, using ddPCR, as previously described [23]. Briefly, for HPV detection, 10 ng of DNA were conducted to Singleplex PCR reactions with 2X ddPCR Supermix for Probes (No dUTP) (BioRad, Hercules, CA, USA), 900 nM of each primer and 300 nM of the probe in a total volume of 22 µL followed by droplet generation using an automated droplet generator (BioRad) according to the optimized protocol [23]. Droplets were generated using a droplet generator (Bio‐Rad). The PCR cycling parameters included 10 min at 95 °C, 50 cycles at 96 °C for 30 s and 61 °C for 30 s, and one cycle at 98 °C for 10 min followed by a 4 °C hold. Droplet fluorescence was assessed using a droplet reader (Bio-Rad). Analysis of the ddPCR data for allele calling and the calculation of absolute copy numbers were performed using QuantaSoft software, version 1.7.4 (Bio‐Rad). Caski (+ HPV 16) and SiHa (-HPV) cell lines were used as controls and were obtained from the ATCC cell bank.

### mRNA expression profiles by nanostring

mRNA expression of a panel of 770 genes associated with 13 cancer-associated canonical pathways related to basic cancer biology, was evaluated using the nCounter PanCancer Pathways Panel, NanoString technology (Nanostring Technologies, USA) for HCB-541 cells and HACAT (immortalized human keratinocyte cell line) cells (used as a non-tumor counterpart) for differential expression analysis, as previously reported [17].

## Results

### Establishment of a primary cutaneous squamous cell carcinoma (cSCC) cell culture

**Fig. 1:**
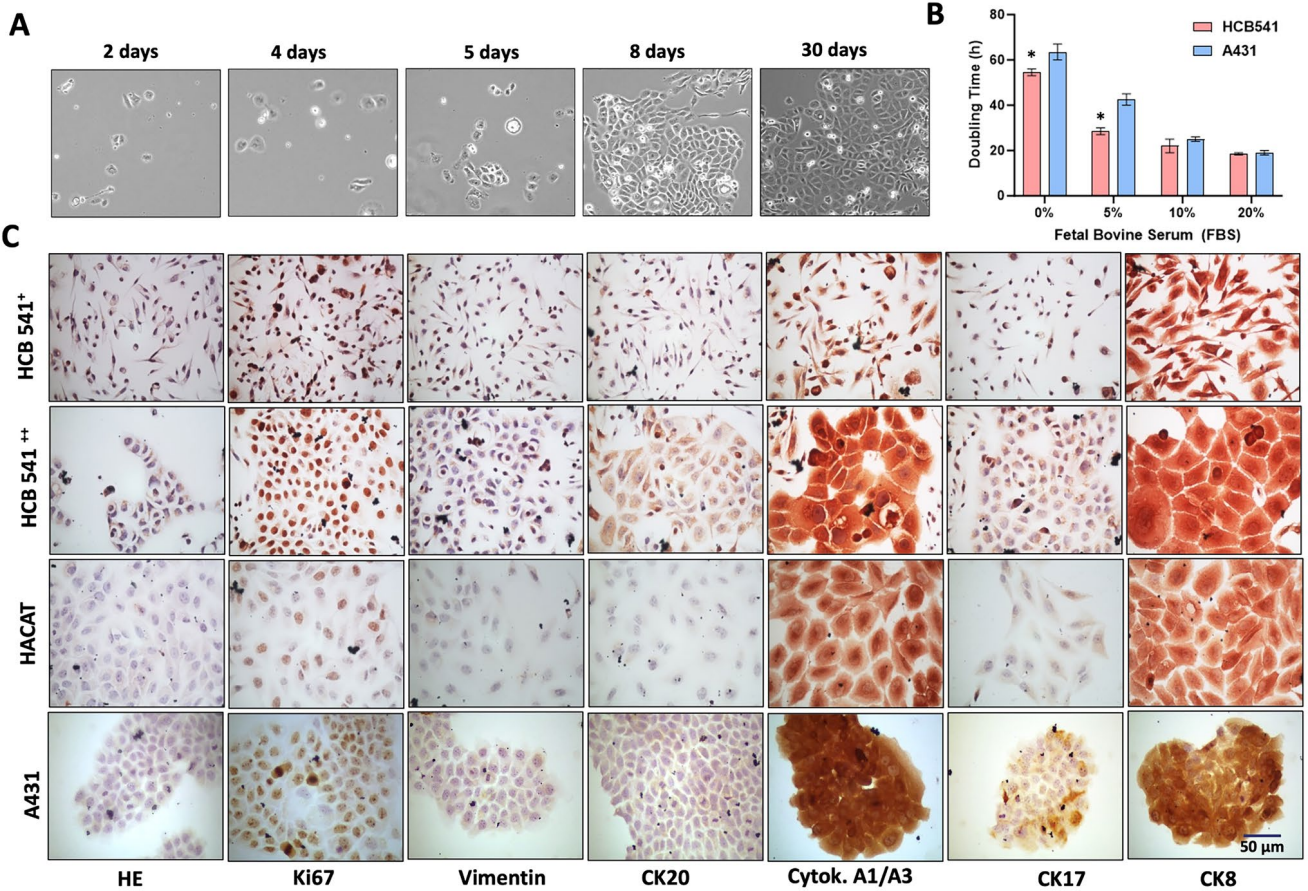
Morphological and proliferative characterization of HCB-541 cell line. **A** Representative photomicrograph of the HCB-541 cell line at 10 × magnification, showing the establishment days. **B** Real-time cell analysis showing the doubling time of HCB-541 cells under different SFB concentrations, with bars representing time (hours) at 60 h. **C** Hematoxylin eosin staining and immunocytochemistry of HCB-541 cells for the expression of Ki67; vimentin and cytokeratin proteins profile (CK20, Cytokeratin A1/A3, CK17, CK8). ^+^Early-passage number (< 5); ^++^ Late-passage number (> 20). Photomicrographs at × 100 magnification. *, ** represents intergroup statistical differences (*p* < 0.05)

A tumor biopsy in the region of the left armpit (Supplementary Fig. 1), was performed for anatomopathological analysis confirming the diagnosis of cutaneous squamous cell carcinoma (cSCC). Surgical specimens measuring 1.5 × 1.3 cm^2^, were collected by sterile scalpels in sterile tubes containing 5 mL of DMEM serum-free medium, supplemented with 1% penicillin–streptomycin. After cells disaggregate protocol two populations were observed, then over 15 days, showed a clustered cell group with fibroblastic morphology that were removed by differential trypsinizations steps to enrich the keratinocytes population. In the initial passages, the growth of the cell was observed in disconnected blotches, after several passages the cell lines acquired a similar keratinocytes behavior (Fig. 1A).

Different growing conditions were done to analyze the HCB-541 growth rates by real-time protocol using xCELLigence RTCA systems. We consider the culture growth rate doubling time between 19 and 22 h, exposed to 10% or superior FBS concentrations and greater than 24 h at concentrations less than 10% FBS concentrations, and without SFB, the HCB-541 cell line obtained a cell index of more than 50 h (Fig. 1B).

HPV infection was not detected in both patient's tumor and HCB-541 cell line (Supplementary Fig. 2).

### Cell line characterization by immunohistochemistry and immunocytochemistry

Hematoxylin and eosin staining of the tumor biopsy and HCB-541 cell line showed similar profile, with clear cytoplasm and large, basophilic nuclei with visible nucleoli and a variable number of mitoses (Fig. 1C). HCB-541 cell depicted a weak vimentin staining, strong staining of Ki67, cytokeratin 20, cytokeratin A1/A3, cytokeratin 17 and cytokeratin 8 markers detected in early and late passages (Fig. 1C). A similar expression profile was observed on HACAT (immortalized human keratinocytes) and A431 (epidermoid carcinoma cell line) (Fig. 1C).

**Fig. 2:**
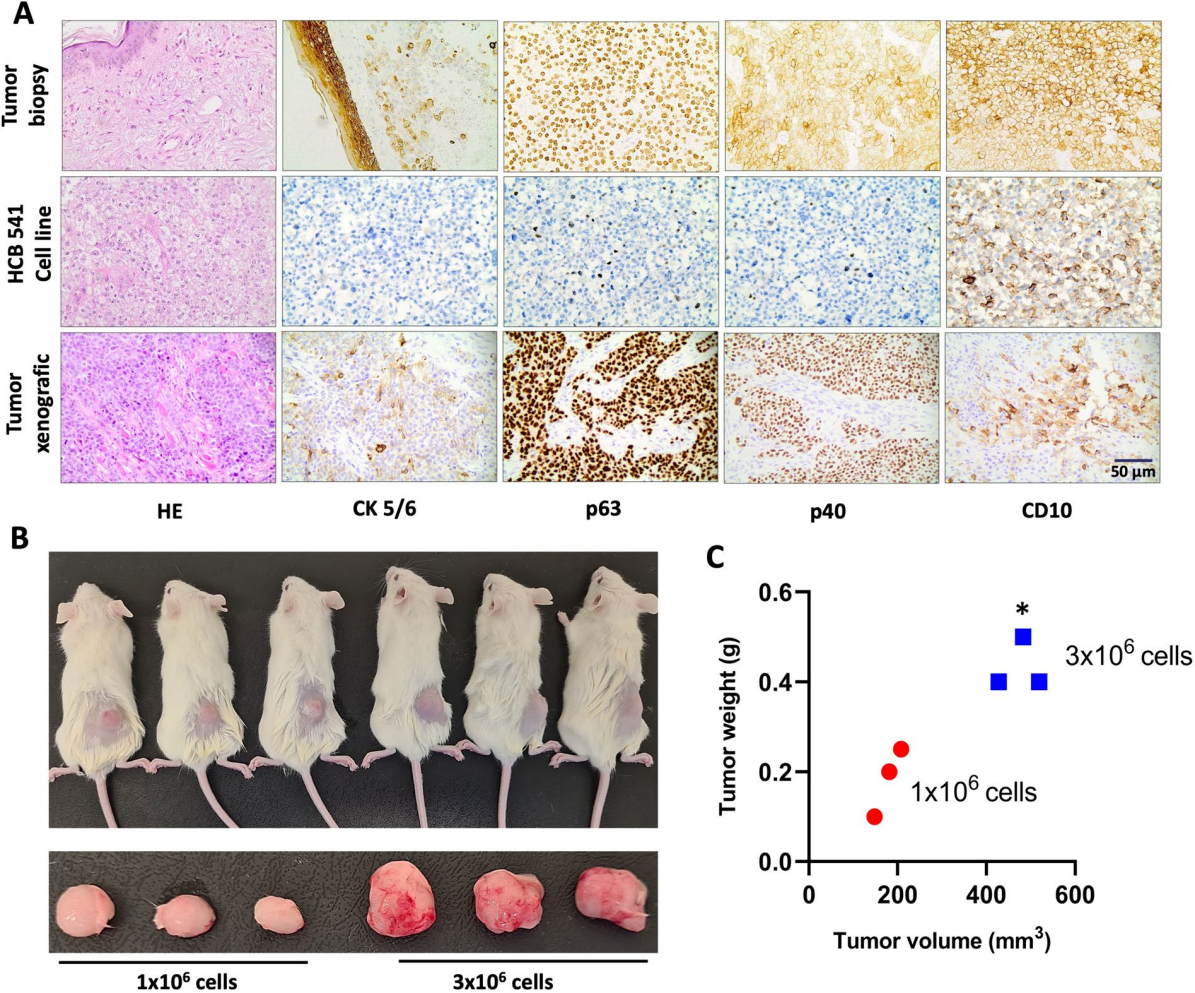
Immunohistochemical staining for comparative expression among the patient's tumor, HCB-541 cell line, and xenograft model. **A** Photomicrography at 100 × magnification HCB-541 passage number: 20. The protein markers studied were Cytokeratin 5/6 (CK5/6), protein 63 (p63), protein 40 (p40), and Common Acute Lymphoblastic Leukemia Antigen (CD10). **B** Subcutaneous inoculation of 1 × 10^6^ (*n* = 3) and 3 × 10^6^ (*n* = 3) cells resulted in tumor growth after 2 weeks in NSG mice. **C** Tumor volume versus tumor weight of the two amounts 1 × 10^6^ (*n* = 3) and 3 × 10.^6^ (*n* = 3) cells that were inoculated subcutaneously. * represents intergroup statistical differences (*p* < 0.05)

Positive immunostaining for the CK5/6, p63, p40 and CD10 antibodies was exclusively evident in the tumor biopsy and xenograft model (Fig. 2A). We observed that only CD10 positivity was conserved in the HCB-541 cell line and also in the xenograft model (Fig. 2A). Positive p63 immunohistochemical staining was exclusively discerned within the tumor biopsy and xenograft model (Fig. 2A).

### Cell line authentication and mycoplasma contamination test

The HCB-541 cell line was mycoplasma free. Short tandem repeat fingerprinting was performed using DNA from the cell line and tumoral biopsy. We detected the loss of one allele from D5S818 and TPOX regions after the establishment of the culture, and the HCB-541 cell line showed at least 90% identical short tandem repeats compared with patient’s biopsy (Supplementary Table 3).

### In vivo* tumorigenicity of HCB-541*

The immunohistochemistry analysis indicated that the xenografts replicated the histopathological features of the cSCC tumor biopsy (Fig. 2A). The tumorigenic potential of HCB-541 was investigated in vivo, by subcutaneously injection of 1.5 × 10^6^ or 3 × 10^6^ cells, into the right flank of female NOD Scid Gamma (NSG) mice and we observed tumor growth after 15 days in all animals tested (Fig. 2B, C). In 15 days, the weight of tumors varied from 0.1 to 0.25 g for mice injected with 1.5 × 10^6^ cells and 0.4–0.5 g for mice injected with 3 × 10^6^ cells (Fig. 2C).

### Chromosome analysis

We found two different populations in HCB-541 cell culture, both presenting a composite karyotype. The first was observed in 12 of 20 metaphases, a hypodiploid karyotype, with 40–45 chromosomes, presenting nullisomy of Y, and monosomy of chromosomes 3, 4, 5, 8, 11 and 21 (supplementary Fig. 3A). In addition, we found a partial deletion of 3p, additional material of 5q and two copies of a chromosome 22 with additional material of unknown origin attached in the short arm, as well as a marker chromosome (supplementary Fig. 3A). In the second population (8 of 20 metaphases), a hypotetraploidy karyotype was detected, presenting 81 to 88 chromosomes (supplementary Fig. 3B). We observed the presence of additional material in the 3q, 15q and 22p, disomy of 8, trisomy of 4, 5, 9, 11, 14, 15, 16, 17, 22 and pentasomy of 7, 19, 20. Also, the presence of an isochromosome of 21q, and 2 to 4 marker chromosomes (supplementary Fig. 3B).

According to International System for Human Cytogenomic Nomenclature, the representative karyotype was 40–45,X,−Y, −3,del(3)(p21),−4,−5,add(5)(q13),−8,−11,−21,add(22)(p13) × 2, + mar[cp12]/81–88,XXYY,add(3)(q27) × 2,−4,−5, + 7,−8,−8,−9,−11, + 12,−14,−15,add(15)(q22),−16,−17, + 19, + 20,i(21)(q10) × 2,add(22)(p13) × 2, + 2–4mar[cp8].

### Mutational analysis

**Fig. 3:**
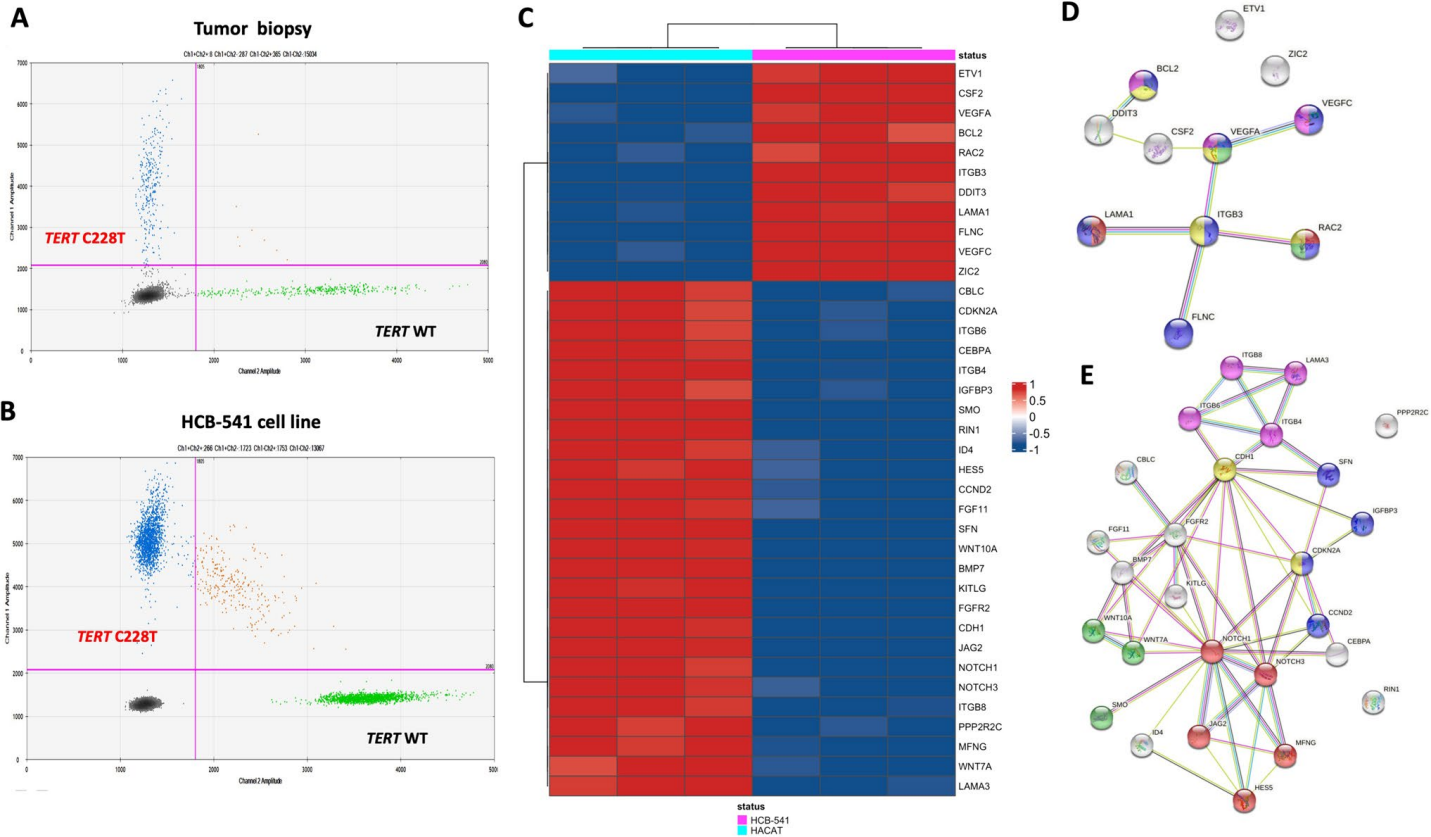
TERT promoter mutation analysis, and gene expression analysis of a pan-cancer panel by NanoString™ and functional pathways analysis **A** *TERT* promoter gene analysis from patient’s tumor. **B** *TERT* promoter gene analysis from HCB-541 tumor cell line after establishment. **C** Heatmap of genes altered in HCB-541 compared with normal HACAT cells. **D** Upregulated gene in silico interaction network. **E** Downregulated gene in silico interaction network. Each circle represents a gene (node), and each connection represents a direct or indirect connection (edge). HCB-541 passage number: 25

Mutation analysis in both patient´s tumor biopsy and cell line revealed 11 variants commun (Table 1). Among the variants identified the ones at *HRAS* p.(Gln61His), *TERT* (1295228 C > T (termed TERT C228T)), and *TP53* p.(Arg248Leu), were classified as pathogenic or likely-pathogenic. We also found mutation only in the tumor tissue in the genes *DICER*, *ESR1*, *KIT*, *PDGFRA*, and *RAF1* (Table 1). Finally, one variant at *NOTCH1* was detected only in the cell line. (Table 1). The hotspot *TERT* promoter mutation (C228T), was further confirmed by ddPCR in both patient’s tumor biopsy and in the HCB-541 cell line (Fig. 3 A, B).

Any gene fusion was identified in the HCB-541 cell line.

Of notice, the MSI analysis revealed that both the HCB-541 cell line and primary tumor tissue presented alteration of BAT26, NR27, BAT25, and NR24 markers, leading to an MSI-High phenotype (Supplementary Fig. 4).

### mRNA expression analysis

We detected the mRNA expression profile of cancer-related genes of the HCB-541 cell line and compared it with the HACAT (non-tumor skin keratinocyte) profile. We found 37 genes differentially expressed in HCB-541 (Fig. 3C and supplementary Table 4). The most remarkable fold change was the overexpression of *ITGB3*, *CSF2*, *LAMA1*, *ETV1*, *ZIC2*, *FLN*C, and *VEGFC* genes in HCB-541 compared with HACAT. At variance, *BMP7*, *WNT10A*, *IGFBP3*, *CEBPA*, *CDH1*, *NOTCH3*, *ID4*, *NOTCH1*, *FGFR2*, *HES5*, *FGF11*, *JAG2*, *ITGB6*, *KITLG*, *PPP2R2C*, *SMO*, *SFN*, *ITGB8*, *CBLC*, *CCND2*, *ITGB4* and *EFNA2* genes were downreguled in HCB-541 cell line, compared with HACAT cell line.

The in silico interaction network analysis using these 11 upregulated genes in the HCB-541 cells showed a tight network of PPI (protein–protein interaction) pathway enrichment (*p* = 0.00497) and functional enrichments suggesting interaction with viral myocarditis (red color), focal adhesion (blue color), VEGF signaling (green color), fluid shear stress and atherosclerosis (yellow color) and AGE-RAGE signaling pathway in diabetic complications process (pink color) (Fig. 3D). The same in silico interaction network analysis of the 26 downregulated genes PPI enrichment of *p-value* of 1.0e-16 and functional enrichments suggest interaction with Notch (red color), p53 signaling (blue color), Basal cell carcinoma (green color), Bladder cancer (yellow color) and ECM-receptor interactions (pink color) (Fig. 3F).

### Protein expression

**Fig. 4:**
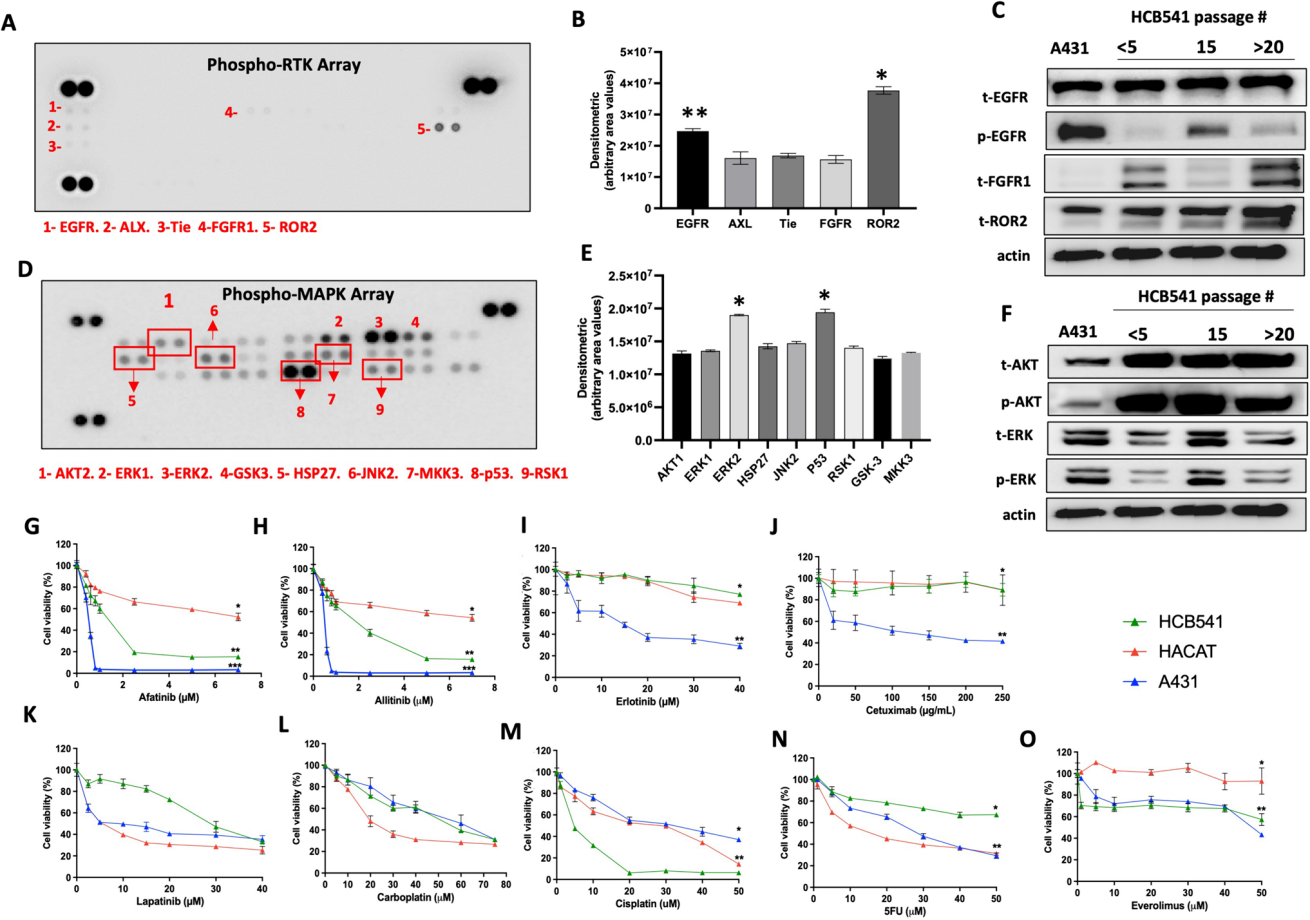
Protein profile of MAPK and RTK phosphorylated of HCB-541 cell line and sensibility to different antineoplastic agents. **A** RTK phosphorylated array of HCB-541 cell line; **B** Densitometric analyses of RTK detection array are represented by bar graphs; **C** RTK phosphorylated profile of HCB-541 by Western blot. **D** MAPK phosphorylated array of HCB-541 cell line. **E** Densitometric analyses of MAPK detection array are represented by bar graphs. **F** MAPK phosphorylated profile of HCB-541 by Western blot analysis. Each RTK and MAPK is duplicate in the arrays (two spots side by side), and three pairs of phosphotyrosine positive controls are in the corners of each array; **G** Afatinib–target-PanErbB, *EGFR* T90M; **H** Allitinib–anti-PanErbB, *EGFR* T90M; **I** Erlotinib–anti-*EGFR* L858R; **D** Cetuximab–monoclonal antibody against EGFR; **K** Lapatinib–anti-EGFR and HER2; **L** Carboplatin–DNA synthesis inhibitor; **M** Cisplatin–DNA synthesis inhibitor; **N** 5-Flouracil–DNA/RNA synthesis inhibitor; **O** Everolimus–mTOR inhibitor. Data presented as the mean of three independent experiments. *, **, *** represents intergroup statistical differences (*p* < 0.05). HCB-541 passage number: 25

Under basal conditions, we detected high phosphorylation levels of EGFR, AXL, Tie, FGFR and ROR2 (Fig. 4A, B). EGFR protein expression was associated with the passage number, in which we detected lower phosphor-EGFR protein levels in higher passages, compared with long culture time (> 20 passage). Contrarily, FGFR1 and ROR2 protein expression levels were higher in the late passage. Among the tested receptors, ROR2 showed higher levels of phosphorylation (Fig. 4C). MAPK protein expression showed several MAPK phosphorylated protein expressions such as AKT1, ERK1/2, GSK-3, HSP27, JNK2, MKK3, P53 and RSK1 (Fig. 4D). We detected a discrete ERK decrease in the late passage compare with initial, and HCB-541 cell line after 20 passages (Fig. 4E, F). A431 cell line was used as a model human cell line of epidermoid carcinoma.

### Chemo-sensitivity profile of HCB-541 cell line

To assess the chemo-sensitivity profile, we exposed HCB-541 to nine different antineoplastic agents. Among the anti-EGFR tested agents, we found IC50 values for afatinib (1.2 ± 0.06 μM) (Fig. 4G), allitinib (1.8 ± 0.32 μM) (Fig. 4H), erlotinib (not founded) and lapatinib (32.8 ± 5.20 μM) inhibitors (Fig. 4K). In addition, the cytotoxic agent such as Carboplatin (48.0 ± 1.24 μM), and Cisplatin (5.1 ± 0.64 μM) demonstrated higher antitumoral effect in HCB-541 cell (Fig. 4L, M). No sensitivity profile could be found for cetuximab, 5-FU and everolimus, displays undetectable IC_50_ values (Fig. 4J–N, O). The A431 tumor cell line exhibited lower IC_50_ values than the HCB-541 cell line only for the EGFR family of afatinib (0.49 ± 0.3 μM), allitinib (0.37 ± 0.9 μM), erlotinib (13.49 ± 0.3 μM), and cetuximab (101.0 ± 0.7 µg/mL), due to its overexpression of this receptor tyrosine kinase. The non-tumor cell line, HACAT, demonstrated sensitivity to treatment with DNA alkylating agents, such as carboplatin (22.45 ± 0.5), cisplatin (18.89 ± 0.6 μM), and the pyrimidine analogue 5-FU (16.69 ± 0.3 μM). In these studies, minimal sensitivity to the other targeted therapies was observed in the HACAT cells used as a control (Fig. 4G–O and Supplementary Table 1).

## Discussion

Advanced cutaneous squamous cell carcinoma (cSCC) is a challenging tumor type that does not respond to surgery or radiotherapy and represents a rare subgroup of cSCC. Currently, only one cSCC cell line (A431) is commercially available, and few studies described metastatic cSCC lines (UT-SCC-7, 59A, and 115), but their molecular profiles have not been reported [24, 25]. In the present study, we established a novel cSCC cell line, HCB-541, from a 67-year-old male patient with advanced squamous cell carcinoma in the armpit.

We used a combination protocol connecting physical and enzymatic tissue digestion to establish the HCB-541 cell line. A recent study reported three decades of work, leading to a panel of cutaneous SCC cell lines [26]. The authors described 16 cell lines derived from 11 patients and reported a long-time interval (10 to 30 years) for the development of these cell lines. Our cSCC cell line, HCB-541, showed a very fast growth, and the formation of tumor in xenograft only after 15 days of cell injection into the mice, suggesting a very aggressive behavior of the HCB-541 cell line.

We found that in supplemented conditions of 5 or 10% FBS, the HCB-541 cell line displayed a doubling time of about 19–22 h, respectively, similar to the 18–24 h doubling time of A431 cells [27]. The establishment of primary cell cultures is troublesome and time-consuming, and in addition, it has high fibroblastic and microbiologic contamination rates [28]. We eliminated fibroblastic contamination from HCB-541 by using a fast trypsinization protocol after higher passage. After fibroblastic exclusion, the cytokeratin profile showed a stronger immunostaining for pan-cytokeratin A1/A2, and Ki67, which are present in epithelial cells and carcinomas [29]. Interestingly, the HCB-541 cell line had positive staining for cytokeratin 20, which is commonly used for the diagnosis of Merkel cell carcinoma [30] and metastatic salivary gland tumors [31]. After the cell line establishment, only CD10 immunostaining was preserved in the HCB-541 cell line. Some studies have already associated CD10 overexpression with other tumor types, such as SCC [32], cutaneous basal cell carcinoma (BCC) [33].

The karyotype analysis was conducted on the HCB-541 cell line after the twentieth passage. Specifically, 12 out of 20 metaphases exhibited a hypodiploid karyotype, while the remaining 8 out of 20 displayed a hypotetraploid karyotype. The karyotype of HCB-541 cells showed heterogeneous chromosomal populations, which is common in solid tumors [34]. The identification of a hyperdiploid karyotype, characterized by its complexity and the presence of multiple cell populations, has been documented in various primary cell culture establishment, including breast tumor cell lines [35] and prostate models [36]. This phenomenon is attributed to the emergence of cell subclones throughout the tumor's evolutionary process. To enhance our understanding, future investigations employing single-cell RNA sequencing analysis on the HCB-541 cell line will be performed for more precise insights into its cellular heterogeneity.

Our mutation analysis identified the presence of mutations in several cancer-related genes previously reported to be important in cutaneous squamous cell carcinoma [9, 37]. The pathogenic *TP53* variant p.(Arg248Leu) was observed in both tumor tissue and the HCB-541 cell line. This variant is considered highly pathogenic and has been reported in 16 morphological tumor types, including squamous cell carcinoma, glioblastoma, adenocarcinoma, hepatocellular carcinoma, and transitional cell carcinoma [38]. Interestingly, this variant has not been reported in metastatic cSCC before. We also the hotspot detected the promoter *TERT* C228T mutation in both the patient's metastatic tumor and HCB-541 cell line by NGS panel and further confirmed by droplet digital PCR (ddPCR). This mutation has been associated with a poorer prognosis and increased risk of lymph node metastasis in cSCC patients [37]). However, only a few studies have analyzed cSCC and revealed rates varying from 34.7% in invasive stages to no more than 19.4% in in situ cSCC [39]. Another important mutated oncogene identified was *HRAS*, with the presence of a p.(Gln61His). This *HRAS* variant is reported to be mutated in this tumor type and is associated with increased proliferation upon Vemurafenib exposure [40]. Due to the lack of normal tissue, we were unable to confirm the somatic nature of the mutations identified, yet several studies suggest that these oncogenic alterations are somatic [41, 42].

We did not find any gene rearrangement in the HCB-541 cell line. This is in line with the literature that shows similar observation, with the exception of the *ADCK4-NUMBL* recurrent gene fusion, which is not present in our NGS assay [43]. Importantly, we found that both the tumor and HCB-541 cell line exhibited an MSI phenotype. The data on MSI is less explored and conflicting. Some studies reported that MSI is present in a small subset of cases (~ 2%) with invasive or metastatic behavior [44]. This finding is very relevant since it is well-known that MSI is an agnostic biomarker of immunotherapy response [45].

Infection with human papillomavirus (HPV) of the beta genus (betaHPV), which includes subtypes 16 and 18 considered high-risk, is seldom linked to the onset of cutaneous squamous cell carcinoma (cSCC). However, it can contribute to skin cancer development in specific cases, such as patients with the genetic disorder Epidermodysplasia verruciformis (EV) or those who are immunocompromised [46]. Since the association between beta HPV and cutaneous SCC remains unclear [47], we analyzed its presence in the HCB-541 cell line and observed the absence of subtypes 16 and 18.

Protein enrichment pathway analysis revealed a strong correlation with other disorders, such as viral myocarditis, atherosclerosis, and diabetic complications. Interestingly, the patient had a history of two myocardial infarctions prior to the development of cutaneous squamous carcinoma. Downregulated genes in HCB-541 were associated with complex network pathways, such as Notch, which has been implicated in the carcinogenesis process of squamous epithelial malignancies [48]. The tyrosine kinase receptor ROR2 has been reported as upregulated gene in numerous types of human cancer, such as laryngeal squamous cell carcinoma, and endometrial cancer [49, 50]. For the first time, we observed higher expression of the tyrosine kinase receptor ROR2 in a metastatic cutaneous squamous carcinoma cell line.

Our investigation has unveiled significant therapeutic responses with second-generation EGFR inhibitors (afatinib and allitinib), which selectively target multiple members of the ErbB receptor family. We hypothesize that the efficiency of these inhibitors was achieved by irreversible binding to the tyrosine kinase receptor, as compared to the reversible action promoted by erlotinib and cetuximab agents. HCB-541cell line was also sensitive to DNA alkylating agents.

Concluding, we have established an immortalyzed metastatic cutaneous squamous cell carcinoma (cSCC) cell line, HCB-541, which carries pathogenic mutations of *TP53, HRAS* and *TERT* and shows an MSI-High status. The high tumorigenicity of this cell line is also associated with the overexpression of several tumor markers, including ROR2 and FGFR, and it showed sensitivity to irreversible pan-ErbB inhibitors and DNA alkylating agents. The HCB-541 can constitute a useful preclinical model for investigating cSCC biology and its response to therapeutic targets.

### Supplementary Information

Below is the link to the electronic supplementary material.

Supplementary file1 (DOCX 25 KB)

Tumor located in the region of the left armpit. Supplementary file2 (TIF 133319 KB)

HPV detection by Droplet Digital PCR (ddPCR) of tumor biopsy and HCB-541 cell line. HCB-541 passage number: 25. The Caski cell line was employed as positive control to HPV16 status and SiHA as a positive control to HPV18. Blue dots indicate positive droplets to the targets (HPV16 or HPV18), while black dots indicate negative droplets (no amplification of the targets). Supplementary file3 (TIF 7309 KB)

Karyotype analysis of HCB-541 cells showing representative metaphase cells. (A) HCB-541 hypodiploid cell population (40~45,X,-Y,-3,del(3)(p21),-4,-5,add(5)(q13),-8,-11,-21,add(22)(p13)x2,+mar[cp12]); (B) HCB-541 hypotetraploidy cell population (81~88,XXYY,add(3)(q27)x2,-4,-5,+7,-8,-8,-9,-11,+12,-14,-15,add(15)(q22),-16,-17,+19,+20,i(21)(q10)x2,add(22)(p13)x2,+2~4mar[cp8]). M: marker chromosome. HCB-541 passage number: 15. Supplementary file4 (TIF 1225 KB)

Detection of microsatellite instability of tumor biopsy and HCB-541 cell line of mononucleotide repeat markers BAT25, BAT26, NR21, NR24, NR27, HSP110. HCB-541 passage number: 25. (A). Tumor biopsy; (B) HCB-541 cell line. Gray region represents normal allele frequency range. Red region represents altered allele frequency. Supplementary file5 (TIF 18254 KB)

## Data Availability

The data that support the findings of this study are available on request from the corresponding author.
