# Supplementary material for: Establishment and molecular characterization of HCB-541, a novel and aggressive human cutaneous squamous cell carcinoma cell line
Source: Hum Cell. 2024 Apr 3;37(4):1170–83. doi: 10.1007/s13577-024-01054-1 (PMC11194207; doi:10.1007/s13577-024-01054-1)
Supplement: Supplementary file 1 — Supplementary file1 (DOCX 25 KB) [file 13577_2024_1054_MOESM1_ESM.docx]

**Supplementary Table 1.** IC_50_ values and target of antineoplastic agents tested in HCB-541, A431 and HACAT cell lines.

| **Drug** | **Target** | **IC_50_ values (± SEM)** | | | **Range of drug concentrations** |
| --- | --- | --- | --- | --- | --- |
|  |  | **HCB-541** | **A431** | **HACAT** |  |
| Afatinib | Pan-ErBb | 1.2 ± 0.06 | 0.49 ± 0.3 | 8.14 ± 0.6 | µM |
| Allitinib | Pan-ErBb | 1.8 ± 0.32 | 0.37 ± 0.9 | 9.8 ± 0.2 | µM |
| Erlotinib | EGFR | N.f | 13.49 ± 0.3 | 64.01 ± 0.8 | µM |
| Cetuximab | EGFR | N.f | 101.0 ± 0.7 | N.f | µg/mL |
| Lapatinib | EGFR, HER2 | 32.8 ± 5.2 | 8.54 ± 0.9 | 5.59 ± 0.2 | µM |
| Carboplatin | DNA | 48.0 ± 1.24 | 49.27 ± 0.3 | 22.45 ± 0.5 | µM |
| Cisplatin | DNA | 5.17 ± 0.64 | 29.61 ± 0.7 | 18.89 ± 0.6 | µM |
| 5-FU | DNA | N.f | 27.05 ± 0.1 | 16.69 ± 0.3 | µM |
| Everolimus | mTOR | N.f | N.f | N.f | µM |

N.f: not found

**Supplementary Table 2 –** NGS custom cancer gene panel of 48 genes.

| Gene | NM | Location |
| --- | --- | --- |
| *CCND1* | NM_053056.2 | All protein-coding exons for all transcripts |
| *CDKN2A* | NM_000077.4 | All protein-coding exons for all transcripts |
| *HIST1H3B* | NM_003537.3 | All protein-coding exons for all transcripts |
| *HRAS* | NM_005343.3 | All protein-coding exons for all transcripts |
| *MYC* | NM_002467.4 | All protein-coding exons for all transcripts |
| *PTEN* | NM_000314.6 | All protein-coding exons for all transcripts |
| *RET* | NM_020630.4 | All protein-coding exons for all transcripts |
| *TP53* | NM_000546.5 | All protein-coding exons for all transcripts |
| *AKT1* | NM_005163.2 | Exons 3 |
| *ALK* | NM_004304.4 | Exons 21 to 25 |
| *BRAF* | NM_004333.4 | Exons 11 and 15 |
| *CDK4* | NM_000075.3 | Exons 2 |
| *CTNNB1* | NM_001904.3 | Exons 2 to 5 |
| *DDR2* | NM_006182.2 | Exons 17 |
| *DICER1* | NM_177438.2 | Exons 24 to 26 |
| *EGFR* | NM_005228.4 | Exons 18 to 21 |
| *ERBB2* | NM_001005862.2 | Exons 11, 20, 21 and 23 |
| *ERBB4* | NM_005235.2 | Exons 10 and 12 |
| *ESR1* | NM_000125.3 | Exons 4 to 8 |
| *FBXW7* | NM_033632.3 | Exons 7 to 11 |
| *FGFR1* | NM_015850.3 | Exons 12 and 14 |
| *FGFR2* | NM_000141.4 | Exons 7, 12 and 14 |
| *FGFR3* | NM_000142.4 | Exons 6, 7, 9, and 14 to 16 |
| *FOXL2* | NM_023067.3 | Exon 1 (partial) |
| *GNA11* | NM_002067.4 | Exons 4 to 5 |
| *GNAQ* | NM_002072.4 | Exons 4 to 5 |
| *GNAS* | NM_000516.5 | Exons 8 to 9 |
| *H3F3A* | NM_002107.6 | Exon 2 and 3 |
| *H3F3B* | NM_005324.5 | Exon 2 |
| *IDH1* | NM_005896.3 | Exons 4 |
| *IDH2* | NM_002168.3 | Exons 4 |
| *KIT* | NM_000222.2 | Exons 8 to 13 and 16 to 18 |
| *KRAS* | NM_004985.4 | Exons 2 to 4 |
| *MAP2K1* | NM_002755.3 | Exons 2 to 3 |
| *MET* | NM_001127500.2 | Exons 2 and 14 to 20 |
| *MYOD1* | NM_002478.4 | Exons 1 |
| *NOTCH1* | NM_017617.4 | Exons 26 to 28 and 34 |
| *NRAS* | NM_002524.4 | Exons 2 to 4 |
| *PDGFRA* | NM_006206.5 | Exons 12, 14 and 18 |
| *PIK3CA* | NM_006218.3 | Exons 3, 6, 8, 10 and 21 |
| *POLE* | NM_006231.3 | Exons 9 to 14 |
| *PTPN11* | NM_002834.4 | Exons 3 |
| *RAC1* | NM_018890.3 | Exons 3 |
| *RAF1* | NM_002880.3 | Exons 7, 10 and 12 to 15 |
| *ROS1* | NM_002944.2 | Exons 38 and 41 |
| *SMAD4* | NM_005359.5 | Exons 8 to 12 |
| *SF3B1* | NM_012433.3 | Exons 15 to 17 |
| *TERT* | NM_198253.2 | Promotor region, exons 8, 9 and 13. |

NM: accession number links to the mRNA

**Supplementary Table 3 –** Cell line authentication of HCB-541 by short tandem repeat analysis.

| **STR Regions** | **Tumor** | **HCB-541** |
| --- | --- | --- |
| **D5S818** | 11,12 | 11 |
| **D13S317** | 11 | 11 |
| **D7S820** |  | 10,11 |
| **D16S539** | 11,12 | 11,12 |
| **vWA** | 15,19 | 15,19 |
| **TH01** | 9,9.3 | 9,9.3 |
| **AMEL** | X | X |
| **TPOX** | 8,11 | 6,8,11 |
| **CSF1P0** |  | 11,12 |

**Supplementary Table 4 -** Complete list of 37 genes differentially expressed in HCB-541 by NanoString analysis.

| **Downregulated genes** | **Fold change** | **Quartile** |
| --- | --- | --- |
| BMP7 | -8,8 | 0,0033 |
| WNT10A | -7,4 | 0,0032 |
| IGFBP3 | -7,4 | 0,0068 |
| CEBPA | -7,0 | 0,0063 |
| CDH1 | -7,0 | 0,0067 |
| NOTCH3 | -6,8 | 0,0068 |
| ID4 | -6,5 | 0,0073 |
| NOTCH1 | -6,4 | 0,0064 |
| FGFR2 | -6,0 | 0,0026 |
| HES5 | -5,5 | 0,0068 |
| FGF11 | -4,9 | 0,0081 |
| JAG2 | -4,8 | 0,0043 |
| ITGB6 | -4,8 | 0,0063 |
| KITLG | -4,8 | 0,0063 |
| PPP2R2C | -4,7 | 0,0077 |
| SMO | -4,3 | 0,0032 |
| SFN | -4,3 | 0,0066 |
| ITGB8 | -4,1 | 0,009 |
| CBLC | -4,0 | 0,0063 |
| CCND2 | -3,7 | 0,0063 |
| ITGB4 | -3,0 | 0,0032 |
| CDKN2A | -2,9 | 0,0073 |
| RIN1 | -2,8 | 0,0026 |
| MFNG | -2,7 | 0,0063 |
| WNT7A | -2,6 | 0,0081 |
| LAMA3 | -2,4 | 0,0081 |
| **Upregulated genes** | **Fold change** | **Quartile** |
| ITGB3 | 7,4 | 0,0046 |
| CSF2 | 6,0 | 0,0064 |
| LAMA1 | 5,5 | 0,0037 |
| ETV1 | 4,9 | 0,0081 |
| ZIC2 | 4,3 | 0,0026 |
| FLNC | 4,0 | 0,0032 |
| VEGFC | 3,1 | 0,0093 |
| BCL2 | 2,6 | 0,0093 |
| RAC2 | 2,5 | 0,0072 |
| DDIT3 | 2,3 | 0,0063 |
| VEGFA | 2,0 | 0,0064 |
