# Supplementary figures and images for: Establishment and molecular characterization of HCB-541, a novel and aggressive human cutaneous squamous cell carcinoma cell line
Source: Hum Cell. 2024 Apr 3;37(4):1170–83. doi: 10.1007/s13577-024-01054-1 (PMC11194207; doi:10.1007/s13577-024-01054-1)

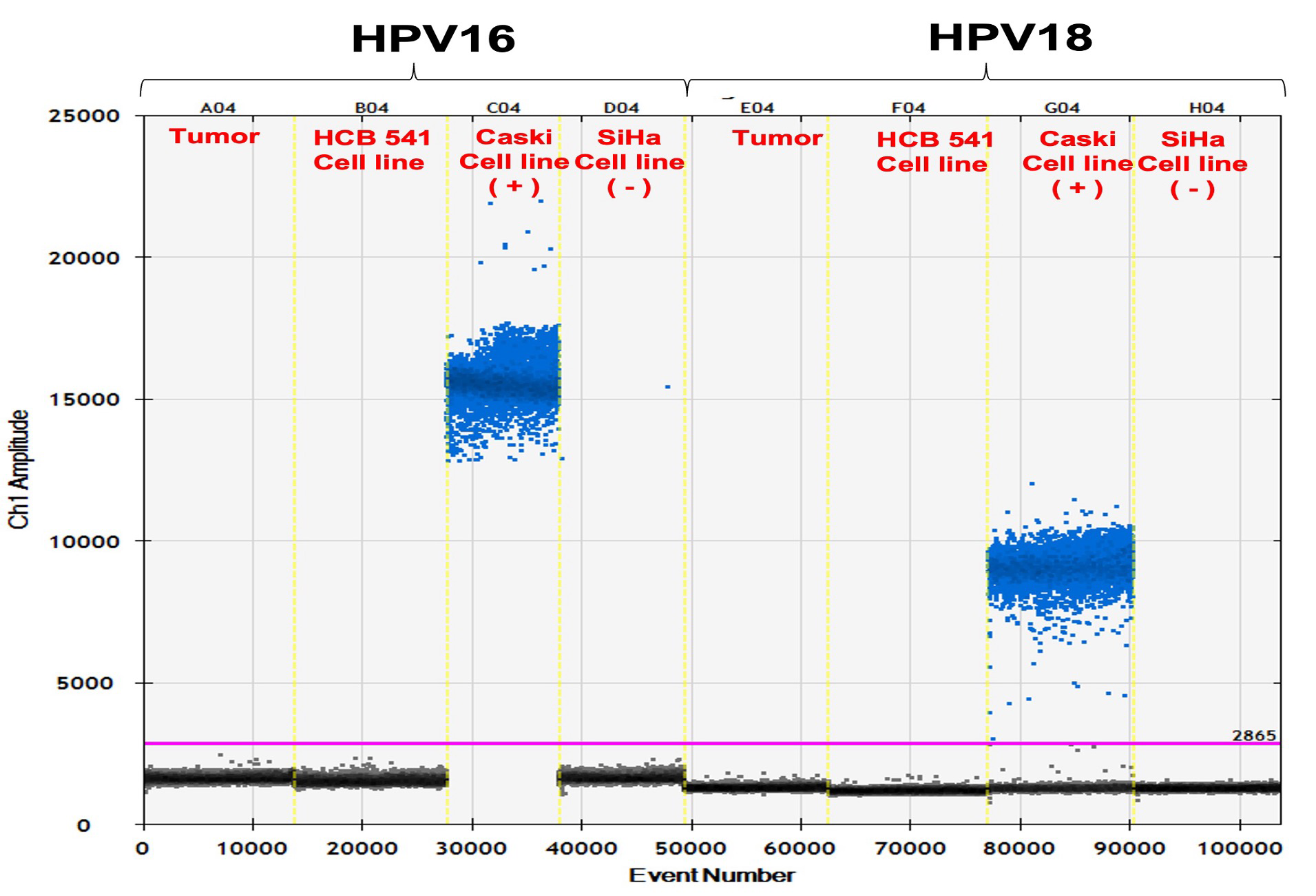

Supplement: Supplementary file 3 — HPV detection by Droplet Digital PCR (ddPCR) of tumor biopsy and HCB-541 cell line. HCB-541 passage number: 25. The Caski cell line was employed as positive control to HPV16 status and SiHA as a positive control to HPV18. Blue dots indicate positive droplets to the targets (HPV16 or HPV18), while black dots indicate negative droplets (no amplification of the targets). Supplementary file3 (TIF 7309 KB) [file 13577_2024_1054_MOESM3_ESM.tif]

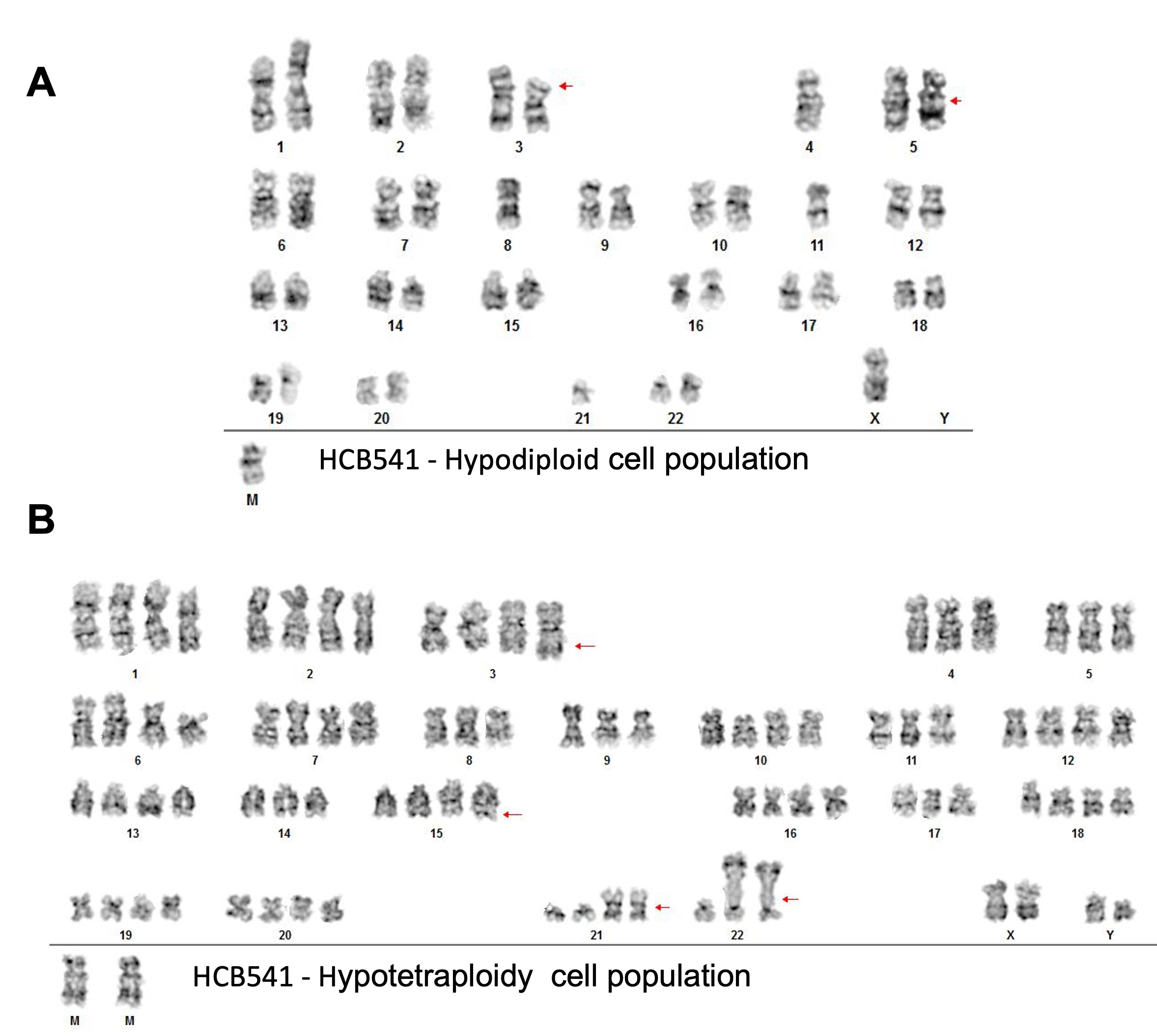

Supplement: Supplementary file 4 — Karyotype analysis of HCB-541 cells showing representative metaphase cells. (A) HCB-541 hypodiploid cell population (40~45,X,-Y,-3,del(3)(p21),-4,-5,add(5)(q13),-8,-11,-21,add(22)(p13)x2,+mar[cp12]); (B) HCB-541 hypotetraploidy cell population (81~88,XXYY,add(3)(q27)x2,-4,-5,+7,-8,-8,-9,-11,+12,-14,-15,add(15)(q22),-16,-17,+19,+20,i(21)(q10)x2,add(22)(p13)x2,+2~4mar[cp8]). M: marker chromosome. HCB-541 passage number: 15. Supplementary file4 (TIF 1225 KB) [file 13577_2024_1054_MOESM4_ESM.tif]

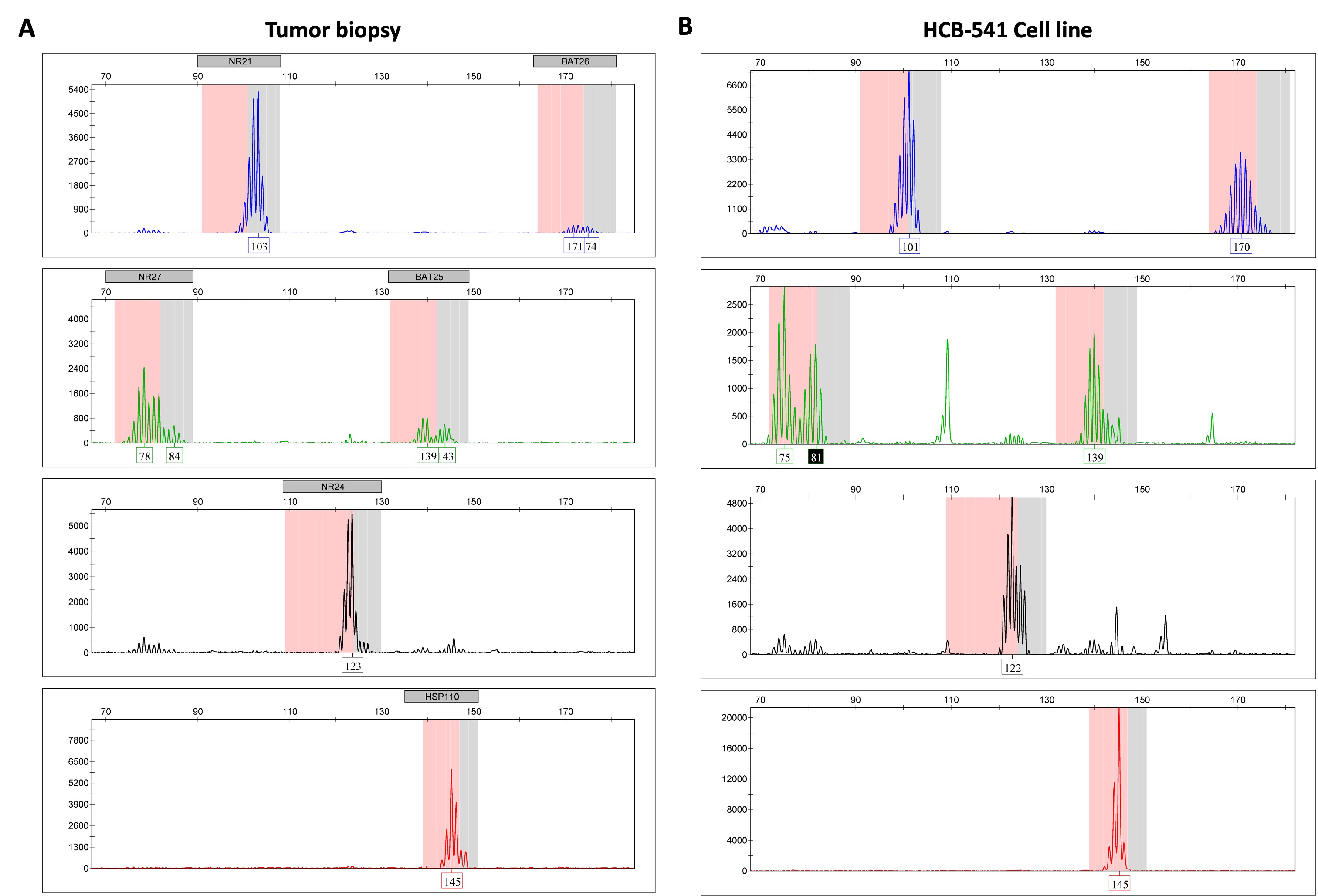

Supplement: Supplementary file 5 — Detection of microsatellite instability of tumor biopsy and HCB-541 cell line of mononucleotide repeat markers BAT25, BAT26, NR21, NR24, NR27, HSP110. HCB-541 passage number: 25. (A). Tumor biopsy; (B) HCB-541 cell line. Gray region represents normal allele frequency range. Red region represents altered allele frequency. Supplementary file5 (TIF 18254 KB) [file 13577_2024_1054_MOESM5_ESM.tif]
